# Coalescence preference in densely packed microbubbles

**DOI:** 10.1038/srep07739

**Published:** 2015-01-13

**Authors:** Yeseul Kim, Su Jin Lim, Bopil Gim, Byung Mook Weon

**Affiliations:** 1 Soft Matter Physics Laboratory, School of Advanced Materials Science and Engineering, SKKU Advanced Institute of Nanotechnology (SAINT), Sungkyunkwan University, Suwon 440-746, Korea; 2 Department of Bio and Brain Engineering, Korea Advanced Institute of Science and Technology (KAIST), Daejeon 305-701, Korea

## Abstract

A bubble merged from two parent bubbles with different size tends to be placed closer to the larger parent. This phenomenon is known as the *coalescence preference*. Here we demonstrate that the coalescence preference can be blocked inside a densely packed cluster of bubbles. We utilized high-speed high-resolution X-ray microscopy to clearly visualize individual coalescence events inside densely packed microbubbles with a local packing fraction of ~40%. The surface energy release theory predicts an exponent of 5 in a relation between the relative coalescence position and the parent size ratio, whereas our observation for coalescence in densely packed microbubbles shows a different exponent of 2. We believe that this result would be important to understand the reality of coalescence dynamics in a variety of packing situations of soft matter.

Coalescence is a common process in soft matter: as examples, bubbles or droplets tend to merge to minimize their surface areas when they are gently in touch. Hydrodynamics and mass transfer are essential in understanding coalescence processes: they determine the driving force and the dynamics. Most studies about coalescence have been focused on neck (contact area) growth dynamics^1^,^2^,^3^,^4^,^5^,^6^,^7^,^8^ that are relevant to corresponding coalescence mechanisms. We recently revealed an interesting coalescence dynamics, named the coalescence preference: a daughter bubble that is merged from two different-sized parent bubbles tends to be placed toward a larger parent (father)^8^. This tendency implies that the rich (father) get richer (closer to daughter). The coalescence preference is attributed to the surface energy release kinetics between two parents and daughter^8^. The universality of the coalescence preference has to be investigated in more various situations and soft matter systems. In this context, an important topic is the coalescence preference in densely packed systems. In fact, bubbles (or droplets) often exist as a form of densely packed clusters in nature, for instance, foams (or emulsions), where coalescence controls their stability and dynamics^9^,^10^,^11^. In densely packed foams, the coalescence events of individual bubbles rapidly take place in a deep position^12^. It is indeed difficult to directly investigate the individual coalescence events inside the densely packed clusters of bubbles in real situations with conventional optical imaging techniques. To clarify the coalescence preference in dense packing, it should be possible to directly distinguish the individual coalescing bubbles from the non-coalescing bubbles in densely packed bubbles. To overcome the technical challenge, X-ray microscopy is appropriate for the coalescence studies for dense packing, particularly thanks to the high penetration capability of X-rays and the excellent phase-contrast enhancement^8^. X-ray imaging technique has been suggested as a powerful technique in soft matter and biomedical studies^13^,^14^,^15^,^16^,^17^,^18^,^19^. X-ray microscopy with spatially coherent X-rays emitted by a synchrotron source enables us to obtain the morphological and the geometrical information of microbubble coalescence with high accuracy^8^,^17^.

Here we report an investigation of the coalescence preference in densely packed microbubbles. We use a high-speed high-resolution X-ray microscopy to clearly visualize the individual coalescence events of densely packed microbubbles with a packing fraction of ~40% inside water. Our results show that the dense packing of bubbles can block the coalescence preference, which is attributed to the confinement effect of a cluster of microbubbles.

## Results

### X-ray imaging of microbubble coalescence

**Figure 1:**
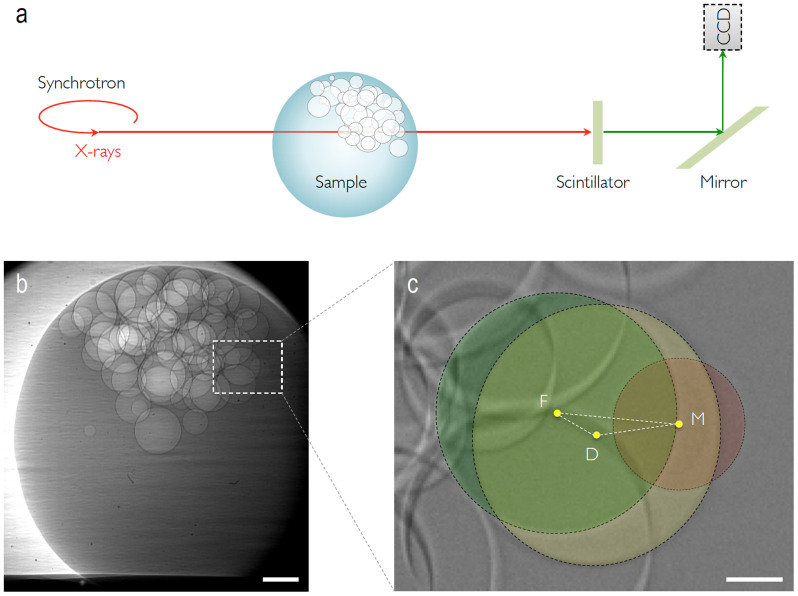
Schematic illustration of X-ray microscopy. (a) Schematic of X-ray microscopy for visualization of the bubble coalescence in a densely packed cluster of microbubbles. (b) Representative X-ray image of the coalescence event in dense packed microbubbles. The scale bar is 200 *μ*m. (c) Analysis method based on the coalescence geometry. The relative position of the merged (daughter: marked by *D*) bubble from the larger (father: marked by *F*) and the smaller (mother: marked by *M*) bubbles is measured through the geometric relation among the side lengths *a* = *FM*, *b* = *DM*, and *c* = *DF*. The scale bar is 50 *μ*m.

To resolve the critical challenge to clearly observe the individual coalescence events of microbubbles inside a liquid medium, we adopted high-speed high-resolution X-ray microscopy. The image acquisition of the coalescence dynamics requires temporal resolution on microsecond scale and spatial resolution on micrometer scale. The conventional optical microscopy is insufficient mainly due to the strong reflection or scattering effects that are usually associated with visible light imaging^17^. As schematically illustrated in Fig. 1(a), we utilized a ultrafast X-ray phase-contrast imaging technique, which combines X-ray phase-contrast microscopy with an ultrafast camera^15^,^17^. The precise separation of the individual coalescence events is enabled by the high penetration capability of X-ray photons at 7–40 keV. Most of all, the exact shape and geometry of the individual bubbles can be clearly measured with X-ray microscopy thanks to the excellent phase-contrast enhancement effect^16^.

A representative image of bubbles inside a water droplet is shown in Fig. 1(b). Here the individual bubbles were initially generated by accident in a water droplet during the drop impact experiments^20^. (A very small amount of salt was included in water for electrical control of shutter and camera^7^,^17^). We obtained two movies (Supplementary Movies 1 and 2) showing totally 12 coalescence events in densely packed clusters of microbubbles. The local packing fraction of microbubbles was measured as ~40% (based on a rough calculation from X-ray movies). These movies provide direct evidence for the usefulness of high-speed high-resolution X-ray microscopy for the clear visualization of coalescing microbubbles within a thick water medium. By direct measurements of the shape and the position (geometry) from the individual coalescence events, we are able to evaluate the individual coalescence preference in densely packed systems.

### Coalescence geometry

A single coalescence event is for example illustrated in Fig. 1(c), which was taken from Fig. 1(b) (from the area marked by the dashed square). The image of Fig. 1(c) was obtained by subtracting an image (just after coalescence) by an image (just before coalescence) with Image-Pro Plus (Media Cybernetics). To describe the relative position of the coalesced bubble, *a_L_* (*a_S_*) is defined as the distance from the larger (smaller) parent center to the closest point on the line linking the parent centers to the position of the merged bubble center. We are able to correctly measure the relative position *a_L_*/*a_S_* by considering a triangle in Fig. 1(c) consisting of the corners that connect the centers of parent bubbles and daughter bubbles. The side lengths of the triangle are then *a* = *FM*, *b* = *DM*, and *c* = *DF*. In turn, two geometric constraints in the triangle of Fig. 1(c) are given: (i) 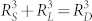 (equivalently *V_M_* + *V_F_* = *V_M_* indicating the volume conservation) and (ii) *a* = *a_S_* + *a_L_*. Here *R* denotes the bubble radius and the subscripts *S* (or *M*), *L* (or *F*), and *D* respectively denote the smaller mother, the larger father, and the merged daughter bubbles. The first constraint comes from the law of mass conservation between before and after coalescence^16^. The mass conservation is equivalent to the volume conservation in a constant density (in our cases, the density is invariant)^16^. The second constraint consists of the lengths *a_S_* = *a* − *a_L_* and *a_L_* = *c* × cos *β* that are determined by the side *c* and the angle *β* (between the sides *a* and *c*). Because of the geometrical condition of cos *β* = (*a*^2^ + *c*^2^ − *b*^2^)/2*ac*, the relative position *a_L_*/*a_S_* can be measured from the side lengths:


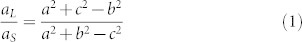


The relative position *a_L_*/*a_S_* is therefore exactly taken by measuring the side lengths of the triangle in Fig. 1(c), which could be achieved by using the high-speed high-resolution X-ray imaging (the same analysis method was introduced in ref. 8).

### Coalescence preference

**Figure 2:**
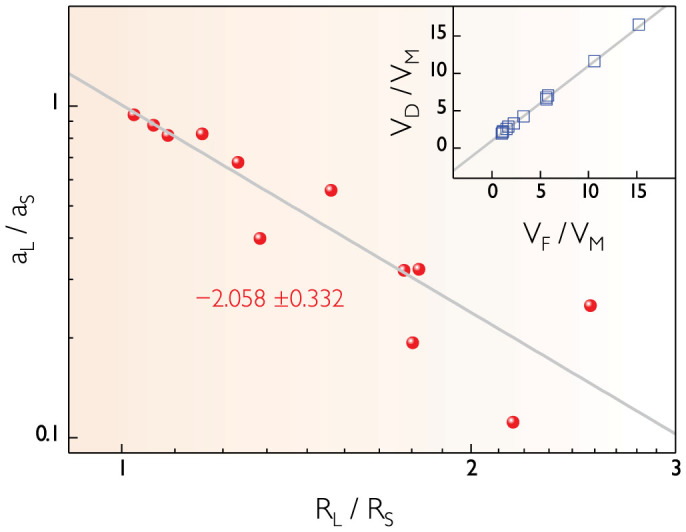
Geometric relation between the relative position (*a_L_*/*a_S_*) and the parent bubble radius ratio (*R_L_*/*R_S_*). The power-law relation as (*a_L_*/*a_S_*) ~ (*R_L_*/*R_S_*)^−*p*^ with the exponent *p* = 2.058 ± 0.332 (fitted by the solid line; the correlation coefficient *R* = −0.89073) confirms that the merged daughter bubble can be placed closer to the larger parent bubble as the parent size difference increases. The inset shows the volume conservation as *V_D_*/*V_M_* = 1 + *V_F_*/*V_M_* or *V_D_* = *V_F_* + *V_M_*: the merged daughter bubble volume (*V_D_*) is equal to the total volume of the parent bubbles (*V_M_* and *V_F_* for smaller mother and larger father bubbles) (the solid line in the inset indicates the theoretical volume conservation).

Interestingly, the relative position of the merged bubble *a_L_*/*a_S_* is dependent upon the parent bubble radius ratio *R_L_*/*R_S_* as illustrated in Fig. 2. This result confirms the coalescence preference in densely packed systems: that is, the merged bubble can be placed closer to the larger bubble as the parent size difference becomes more significant. Here we find that differently from the previous research^8^, the *p* exponent was measured as *p* = 2.058 ± 0.332 from the power-law relation:


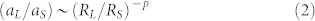


The measured exponent (*p* ~ 2) for densely packed bubbles is much smaller than that (*p* ~ 5) taken for relatively free bubble systems and quite different from that (*p* ~ 3) taken for the center-of-mass theory^8^. Note that the law of mass (volume) conservation holds valid as *V_D_* = *V_F_* + *V_M_*^16^ during the individual coalescence events as highlighted in the inset of Fig. 2. The coalescence preference for free bubbles (*p* ~ 5) was well explained by the surface energy release theory (*p* ~ 5.3 as predicted in theory)^8^. The result for densely packed bubbles in Fig. 2 is unexpected and unexplained yet.

## Discussion

**Figure 3:**
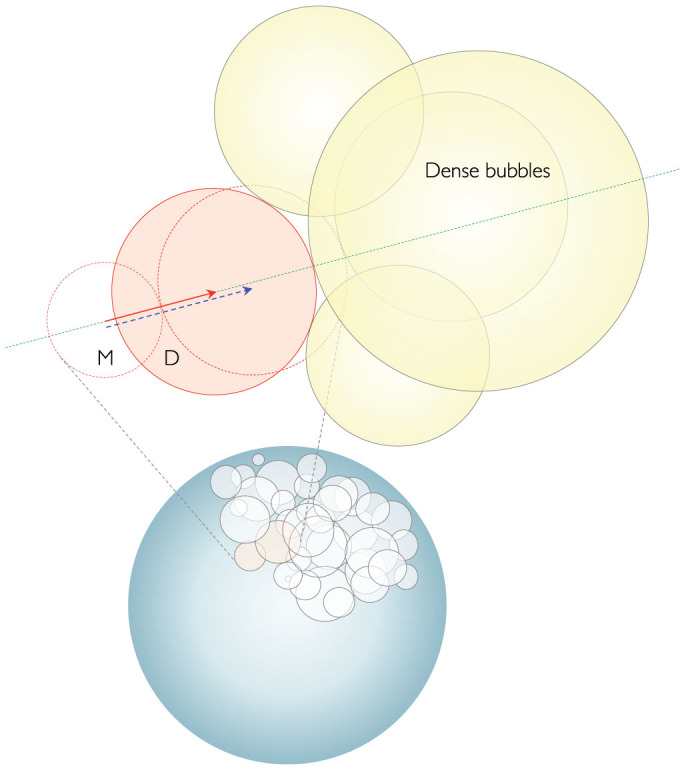
Possibility of the coalescence preference blocked by neighboring bubbles in a densely packed cluster of bubbles. When two parent bubbles (red dotted circles) coalesce into a daughter bubble (marked by *D*), the gas of the mother (marked by *M*) moves toward the father by the Laplace pressure difference (following the blue dashed arrow). The presence of the neighboring bubbles (yellow colored circles) can block the coalescence preference for free bubbles by restricting the final position of the daughter bubble (moving toward the place marked by the red solid arrow).

To reconcile our result (*p* ~ 2 in Fig. 2) with the previous results (*p* ~ 5 in ref 8), we suggest an explanation as follows. As illustrated in Fig. 3, we believe that the coalescence preference between two bubbles would generally take place in a densely packed cluster of microbubbles. The gas of the smaller mother bubble (marked by *M*) tends to move toward the larger father bubble due to the Laplace pressure difference 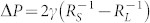 between two parent bubbles (where *γ* is the water surface tension, *γ* = 72 mN/m)^8^. The final position of the merger daughter bubble (marked by *D*) will be determined by the drive of Δ*P* by following the blue dashed arrow in Fig. 3 unless there are no neighboring bubbles. For example, Δ*P* is estimated to be ~720 Pa for *R_S_* = 100 *μ*m and *R_L_* = 200 *μ*m. In a case of the presence of the densely packed bubbles, the motion of the daughter bubble can be blocked by the confinement effect of the neighboring bubbles.

The confinement effect by a cluster of bubbles (or foams) is evaluated by the confinement pressure *P*^21^, which corresponds to the osmotic pressure Π(*ϕ*) as a function of the liquid volume fraction *ϕ*^22^,^23^. At a wet foam condition (*ϕ* ~ 0.1), *P* ≈ 0.5(*γ*/*R*) is estimated to be ~360 Pa for the neighboring bubbles with *R* = 100 *μ*m. Interestingly, the Laplace pressure difference that drives the motion of the merged bubble is comparable to the confinement pressure that may retard the motion of the merged bubble: in fact, Δ*P* is slightly larger than *P*. We note that the local bubble volume fraction is quite low (~40%), where the coalescence preference is likely to occur but identical to the case of free bubbles. As a result, the final position of the merged bubble can be placed less closer to the father bubble as illustrated by the red solid arrow in Fig. 3. We believe that the confinement effect by the neighboring bubbles would be responsible for the *p* exponent to be near 2 for densely packed bubble systems (in our experiments), rather than 5 for free bubble systems (in the previous results^8^).

In conclusion, we present an investigation of the coalescence preference in densely packed microbubbles. Interestingly our results show that the final position of the merged bubble can be placed *less closer* to the larger bubble: the position for dense bubbles can be quite different from that expected for free bubbles that are not confined in space. In particular, high-speed high-resolution X-ray microscopy is much useful to provide the key evidence by taking the individual coalescence events from densely packed microbubbles with a local packing fraction of ~40%. The geometric relation between the relative coalescence position and the parent size ratio clearly demonstrates that the coalescence in densely packed bubbles may have a different exponent of 2, which was observed as 5 for free bubbles and expected to be 5.3 in theory^8^. We believe that this result would be important to understand the reality of coalescence dynamics in a variety of packing situations of soft matter.

## Methods

The X-ray imaging experiments were performed at 32-ID-B and C in the Advanced Photon Source of the Argonne National Laboratory with the experimental setup as schematically illustrated in Fig. 1(a). We used a focused monochromatic X-ray beam at the photon energy of 7–40 keV (resolution Δ*E*/*E* ≈ 0.0001, beam size ≈ 1 × 2 mm^2^). The high brightness yielded spatial resolution (~2 *μ*m per pixel) in a short acquisition time (~185 *μ*s per frame). Unlike the previous research^8^, the key idea of the experimental setup is that the individual bubbles are initially generated by accident in a water droplet during the drop impact experiments^20^. The water droplet has 1.4 mm in radius. After passing through the water droplet, the transmitted X-rays were converted by a scintillator to visible lights, which were then reflected by a mirror and magnified by an objective lens (Mitutoyo M Plan Apo 5, NA = 0.14). After magnification, the image on the scintillator was captured by a CMOS camera (1,024 × 1,024 pixels; Photron SA 1.1, Photron) that was synchronized with the fast rotary stage and a fast shutter. The whole imaging system was carefully aligned to gravity by using a digital inclinometer with 0.001° accuracy.

## Author Contributions

B.M.W. conceived the study and performed the experiment. Y.K. and S.J.L. analyzed the data and calculated the statistical information. B.G. participated in the data interpretation. All authors discussed the results and wrote the manuscript.

## Supplementary Material

Supplementary Information Movie S1

Supplementary Information Movie S2

## References

[b1] Eggers J., Lister J. R. & Stone H. A. Coalescence of liquid drops. J. Fluid Mech. 401, 293–310 (1999).

[b2] Menchaca A., Martinez-Davalos A., Nunez R., Popinet S. & Zaleski S. Coalescence of liquid drops by surface tension. Phys. Rev. E 63, 046309 (2001). 10.1103/PhysRevE.63.046309 11308947

[b3] Aarts D. G. A. L., Lekkerkerker H. N. W., Guo H., Wegdam G. H. & Bonn D. Hydro-dynamics of droplet coalescence. Phys. Rev. Lett. 95, 164503 (2005). 16241805 10.1103/PhysRevLett.95.164503

[b4] Thoroddsen S. T., Etho T. G., Takehara K. & Ootsuka N. On the coalescence speed of bubbles. Phys. Fluids 17, 071703 (2005).

[b5] Bremond N., Domejean H. & Bibette J. Propagation of drop coalescence in a two-dimensional emulsion: A route towards phase inversion. Phys. Rev. Lett. 106, 214502 (2011). 21699303 10.1103/PhysRevLett.106.214502

[b6] Vakarelsk I. U. *et al.* Dynamic interactions between microbubbles in water. Proc. Natl. Acad. Sci. U.S.A. 107, 11177 (2010). 20534552 10.1073/pnas.1005937107 PMC2895070

[b7] Paulsen J. D., Carmigniani R., Kannan A., Burton J. C. & Nagel S. R. Coalescence of bubbles and drops in an outer fluid. Nature Commun. 5, 3182 (2014). 24458225 10.1038/ncomms4182

[b8] Weon B. M. & Je J. H. Coalescence preference depends on size inequality. Phys. Rev. Lett. 108, 224501 (2012). 23003601 10.1103/PhysRevLett.108.224501

[b9] Ganan-Calvo A. M., Fernandez J. M. & Marquez Oliver A. Coarsening of monodisperse wet microfoams. Appl. Phys. Lett. 84, 49894991 (2004).

[b10] Murray B. S. Stabilization of bubbles and foams. Curr. Opin. Colloid Interf. Sci. 12, 232241 (2007).

[b11] Colin A. Coalescence in Foams in Foam Engineering: Fundamentals and Applications (Wiley, United Kingdom, 2012).

[b12] Biance A.-L., Delbos A. & Pitois O. How Topological Rearrangements and liquid fraction control liquid foam Stability. Phys. Rev. Lett. 106, 068301 (2011). 21405499 10.1103/PhysRevLett.106.068301

[b13] Wilkins S. W., Gureyev T. E., Gao D., Pogany A. & Stevenson A. W. Phase-contrast imaging using polychromatic hard X-rays. Nature 384, 335338 (1996).

[b14] Bech M. *et al.* In-vivo dark-field and phase-contrast x-ray imaging. Sci. Rep. 3, 3209 (2013). 24220606 10.1038/srep03209 PMC3826096

[b15] Wang Y. *et al.* Ultrafast X-ray study of dense-liquid-jet flow dynamics using structure-tracking velocimetry. Nature Phys. 4, 305–309 (2008).

[b16] Weon B. M., Je J. H., Hwu Y. & Margaritondo G. A coherent synchrotron X-ray micro-radiology investigation of bubble and droplet coalescence. J. Synchrotron Radiat. 15, 660662 (2008). 10.1107/S0909049508025363 PMC2573953 18955775

[b17] Fezzaa K. & Wang Y. Ultrast X-ray phase-contrast imaging of the initial coalescence phase of two water droplets. Phys. Rev. Lett. 100, 104501 (2008). 18352193 10.1103/PhysRevLett.100.104501

[b18] Lee J. S., Weon B. M. & Je J. H. X-ray phase-contrast imaging of dynamics of complex fluids. J. Phys. D: Appl. Phys. 46, 494006 (2013).

[b19] Jung J. W. *et al.* Four-dimensional visualization of rising microbubbles. Sci. Rep. 4, 5083 (2014). 24866552 10.1038/srep05083 PMC4035580

[b20] Lee J. S., Weon B. M., Je J. H. & Fezzaa K. How does an air film evolve into a bubble during drop impact? Phys. Rev. Lett. 109, 204501 (2012). 23215492 10.1103/PhysRevLett.109.204501

[b21] Lespiat R., Cohen-Addad S. & Höhler R. Jamming and flow of random-close-packed spherical bubbles: an analogy with granular materials. Phys. Rev. Lett. 106, 148302 (2011). 21561226 10.1103/PhysRevLett.106.148302

[b22] Höhler R., Sang Y. Y. C., Lorenceau E. & Cohen-Addad S. Osmotic pressure and structures of monodisperse ordered foam. Langmuir 24, 418–425 (2008). 18067333 10.1021/la702309h

[b23] Maestro A., Drenckhan W., Rioa E. & Höhler R. Liquid dispersions under gravity: volume fraction profile and osmotic pressure. Soft Matter 9, 2531–2540 (2013).

